# Supplementary material for: Nanoassemblies from Amphiphilic Sb Complexes Target Infection Sites in Models of Visceral and Cutaneous Leishmaniases
Source: Pharmaceutics. 2022 Aug 21;14(8):1743. doi: 10.3390/pharmaceutics14081743 (PMC9412331; doi:10.3390/pharmaceutics14081743)
Supplement: Supplementary file 1 [file pharmaceutics-14-01743-s001.zip › pharmaceutics-1839037-supplementary_Corrected.pdf]

pharmaceutics

Supplementary materials

Nanoassemblies from amphiphilic Sb complexes target infection sites in models of visceral and cutaneous leishmaniases, by Juliane S. Lanza, Virgínia M. R. Vallejos, Guilherme S. Ramos, Ana Carolina B. de Oliveira, Cynthia Demicheli, Luis Rivas, Sébastien Pomel, Philippe M. Loiseau and Frédéric Frézard

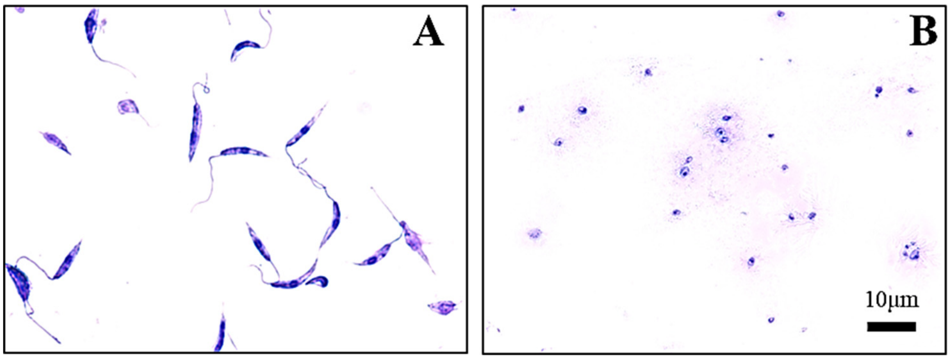

Figure S1. Photomicrographies of axenic *L. donovani* (LV9). In (**A**) promastigotes in log phase (4 days after initial inoculum of 200.000 parasites per mL of new medium) and (**B**) amastigote-like, transformed promastigotes, after 72 h incubation at 37 °C, 5% CO_2 atmosphere. Parasites were fixed with cold methanol (>99%) in pre-coated poly-lysine slides, air-dried and stained for 5 min with Giemsa stain.

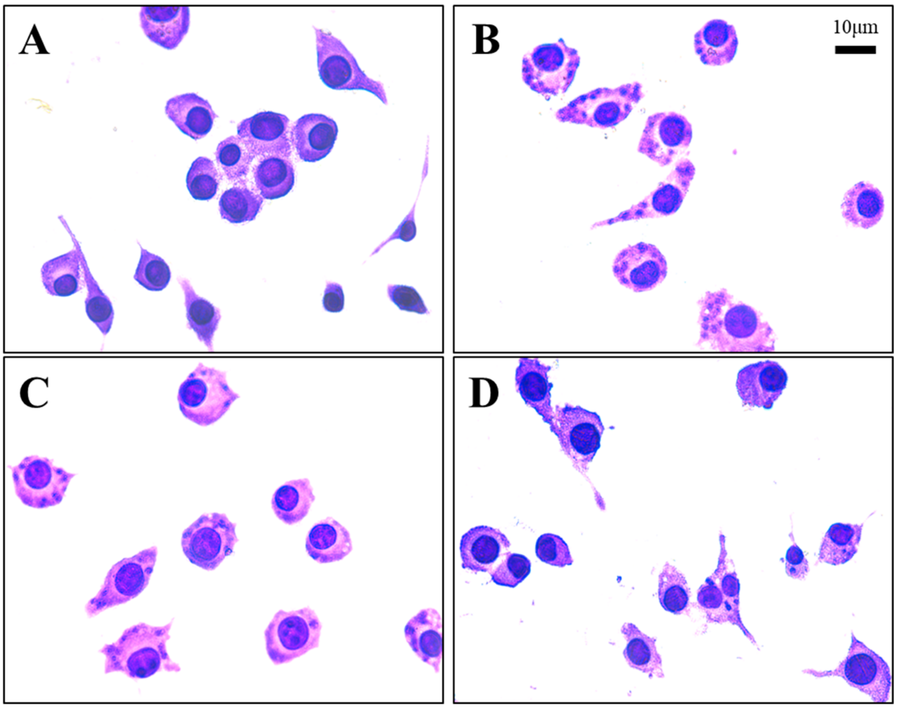

Figure S2. Antileishmanial efficacies of amphiphilic antimony complexes in *L. donovani*-infected RAW264.7 murine macrophages. (**A**) Represents the non-infected control, i.e., RAW264.7 incubated with complete RPMI medium 10% FCS; (**B**) Shows untreated infected macrophages, the rate of infection was higher then 80%; (**C**) Infected macrophages treated with 50 uM of SbL8 showed decrease of infection rate and parasite load per cell, and (**D**) Infected macrophages treated with 50uM of SbL10 showed very few infected cells with low numbers of amastigotes per infected cell.

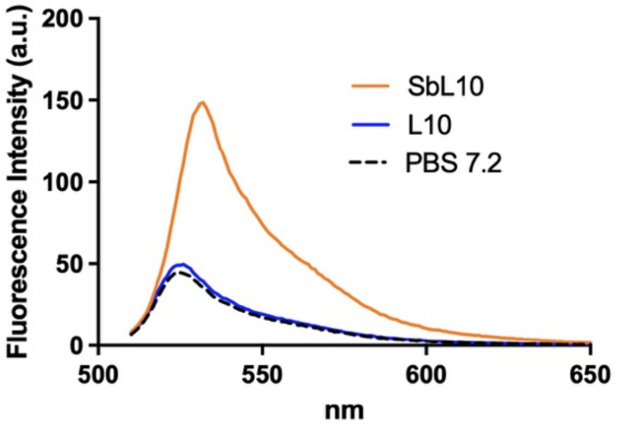

Figure S3. Fluorescence emission spectra of MT-11-BDP (miltefosine fluorescent analog) in saline, SbL10 and L10 suspensions at 1 mM of L10.

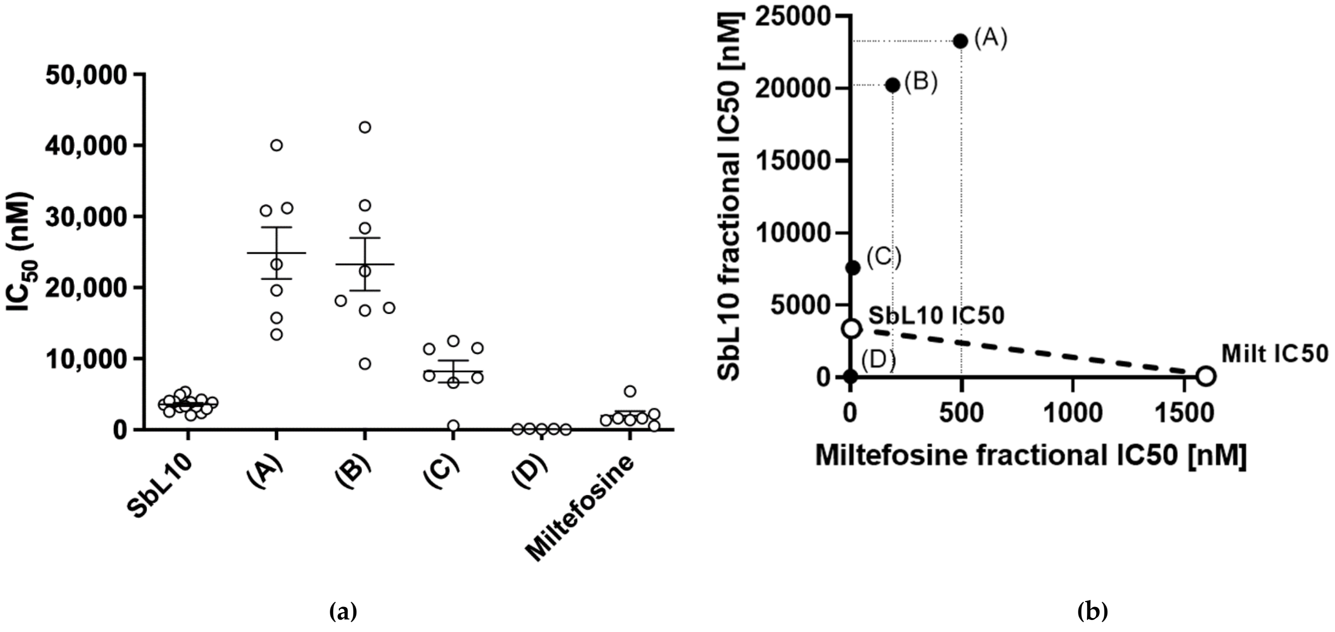

Figure S4. *In vitro* compound's activity interaction between SbL10 and miltefosine on intramacrophage *L. donovani* (LV9). (**a**): IC_{50} of SbL10 alone, miltefosine alone and their combinations at different molar ratio (A–D); error bars indicate standard error of the mean (SEM). (**b**): fixed-ratio isobolograms of fractional drug IC_{50} SbL10-miltefosine partner drug combination at different molar ratios. (A) = 0.8[miltefosine] + 0.2[SbL10]; (B) = 0.6[miltefosine] + 0.4[SbL10]; (C) = 0.4[miltefosine] + 0.6[SbL10]; (D) = 0.2[miltefosine] + 0.8[SbL10]).

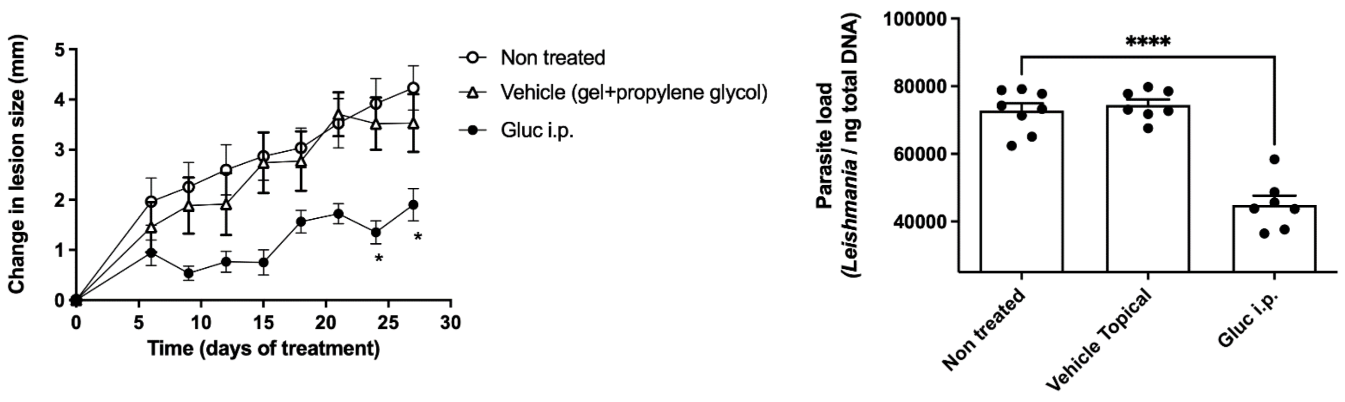

(**a**) (**b**)

Figure S5. Antileishmanial efficacy in murine CL model of i.p. treatment with Glucantime®, in comparison to topical treatment with 1:1(*v*/*v*) water:PG gel and no treatment. BALB/c mice were infected intradermally at the tail base with 1 × 10^6 *L. amazonensis* (MHOM/BR/1989/BA199). After 35 days of infection, animals (n = 7–8 mice per group) were treated daily for 27 days with either 1:1(*v*/*v*) water:PG gel containing 1% (*w*/*v*) hydroxyethyl cellulose (50 uL/application) or Glucantime® by i.p. route at 200 mg/kg/day and compared to non-treated control group. (**a**) Variation of the lesion size with time shown as mean ± SEM. * $p < 0.05$ with respect to control untreated group (Two-way repeated measures ANOVA, followed by Tukey's multiple comparisons test). (**b**) Parasite load as determined by qPCR at the end of treatment (30 days after the start of treatment). **** $p < 0.0001$ for comparison of each treated group with the control group, one-way ANOVA test, followed by Dunnett's multiple comparison post-test. Data are shown as mean + SEM.

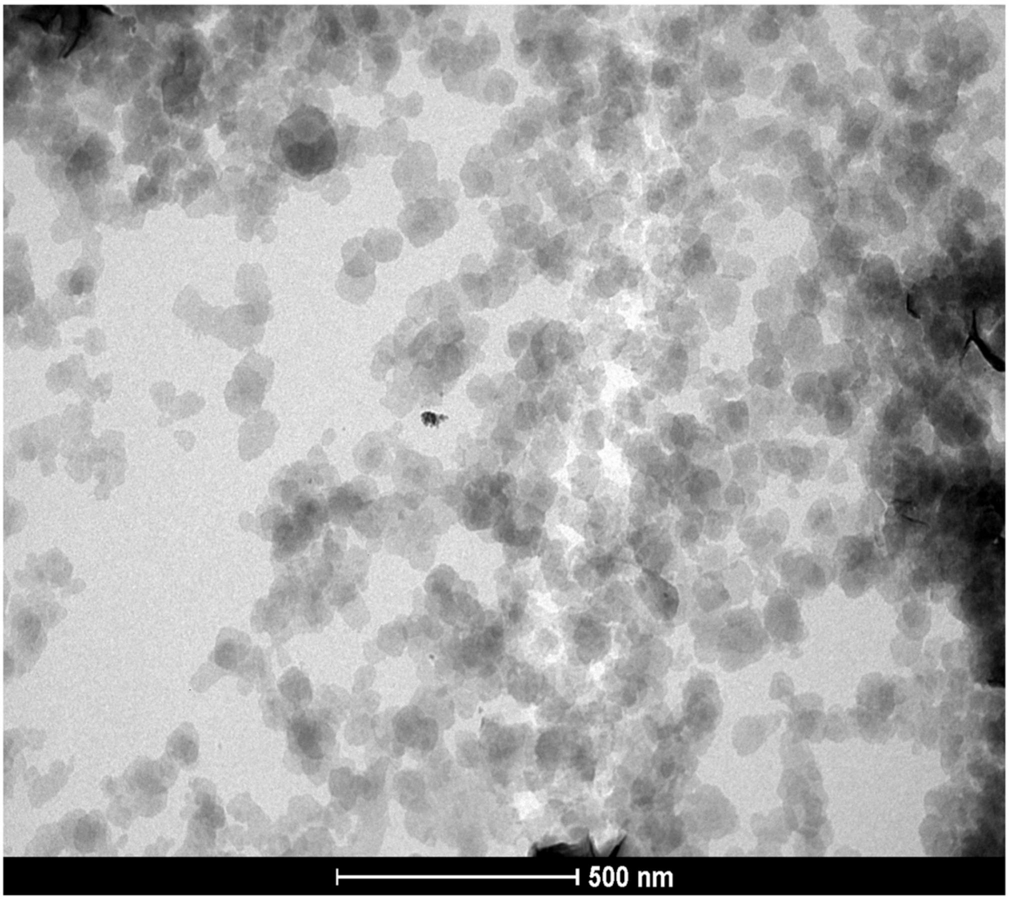

Figure S6: Characterization of the morphology of SbL8 nanoassemblies in PBS 7.2 by transmission electron microscopy.

Table S1. Particle size distribution and zeta potential of nanoparticles in SbL8 and SbL10 suspensions in NaCl 0.15 M (pH 5.5) or HCl 0.05 M.

Sample	Mean hydrodynamic diameter (nm)	Polydispersity index	Zeta potential (mV)
SbL8	93.9	0.281	-27.8
SbL8 + HCl	*	-	-18
SbL10	36.3	0.591	-29.1
SbL10 + HCl	*	-	-16.8

* high polydispersity

Table S2. Individual (IC_{50}) and fractional (ΣFIC) drug inhibitory concentration for the pharmacological interactions between miltefosine and SbL10 towards intracellular *L. donovani* amastigotes, according to modified chequerboard assay

Association (Milt:SbL10 molar ratio)	IC_{50} ± SD [a] of SbL10 (nM)	IC_{50} ± SD [a] of miltefosine (nM)	ΣFIC	Interpretation [b]
-	3242±900	1621±160		
A (4:1)	23285±9500	49.3±30	7.4863	Antagonism
B (3:2)	20232±10500	18.9±10	6.35759	Antagonism
C (2:3)	7592±410	11.9±0.6	2.34908	Additive
D (1:4)	76.5±40	0.3±0.02	0.0238	Synergism

[a]IC_{50} values are expressed in mean inhibitory concentration of 50% infection index (regarding the non-treated control) ± standard deviation. [b]ΣFIC ⩽ 0.5, synergism; ΣFIC > 0.5–4.0, no interaction (or additive); ΣFIC > 4.0, antagonism [15,20,31].

Table S3. Intracellular accumulation of Sb (μg Sb / 10^6 Thp-1 cells) (data from Figure 3a).

Drug	SbL8	SbL10	Gluc
	0.1978	0.19925	0.01055
	0.2635	0.21495	0.014
	0.11995	*1.33055**	0.00015
	0.19445	0.1954	-0.00175
	0.203	0.1938	0.01545
	0.19875	0.16575	*0.1716**
	0.18005	0.187	
	0.16795	0.15975	
	0.2497	0.1645	

* values identified as outliers according to ROUT (Q = 0.1%) and removed for statistical analysis.

Table S4. Accumulation of Sb in the liver of mice (µg Sb / g wet tissue) 24 h after adminitration of different antimonial formulations (data from Figure 3b).

SbL8 i.p.	SbL10 i.p.	Gluc i.p.	SbL8 Oral	SbL10 Oral
84.4677661	69.0254873	8.09595202	13.5832084	62.9835082
183.208396	100.269865	8.54572714	14.197901	61.4242879
111.514243	74.9325337	8.54572714	11.0794603	15.2623688
106.086957	64.1529235	9.44527736	27.9610195	10.6446777
100.734633	99.1154423	4.7976012	10.1949025	42.113943

Table S5. Parasite load in the liver of mice infected with *L. donovani* after different antimonial treatments (data from Figure 5a)

Parasite load (*Leishmania* number per 50 ng of DNA)					
**Non-treated**	**Gluc i.p.**	**SbL8 Oral**	**SbL10 Oral**	**SbL8 i.p.**	**SbL10 i.p.**
12.2	0.00545	6.1	0.427	*0.222**	*2.54**
14.3	0.0263	14.9	0.816	0.0139	0.00584
7.35	0.0166	3.18	0.386	0.0144	0.0124
106	0.00522	0.772	0.52	0.0449	0.01
76.8	0.00756	0.172	1.72	0.0224	0.03
15.5	0.00886	3.5	*8.64**	0.0102	0.02
37.2	0.00376				
28.8	0.0269				
23.3	0.00766				

*values identified as outliers according to ROUT (Q = 0.1%) and removed for statistical analysis.

Table S6. Parasite load in the spleen of mice infected with *L. donovani* after different antimonial treatments (data from Figure 5b)

Parasite load (*Leishmania* number per 50 ng of DNA)					
**Non-treated**	**Gluc i.p.**	**SbL8 Oral**	**SbL10 Oral**	**SbL8 i.p.**	**SbL10 i.p.**
7.77	2.51	2.26	2.76	1.05	0.908
4.97	1.04	2.31	2.31	0.661	0.477
1.35	0.386	2.39	1.19	0.525	0.351
3.31	1.52	2.94	2.43	0.686	0.587
4.08	0.635	2.65	3.77	0.638	0.641
3.75	0.676	2.31	2.25	0.254	0.747
3.87	0.689				
5.44	0.661				
1.42	0.519				

Table S7. Variation of lesion size of mice infected with *L. amazonensis* after different antimonial treatments (data from Figure 6a)

Variation of lesion size with respect to time zero (mm)						
			Non-treated			
Time (day)						
5	0.2	0.1	0.3	0.3	0.3	0.3
10	0.9	0.85	0.85	1.75	0.7	1.2
15	1.05	0.4	1.15	2.65	1.1	1.8
20	1.75	1.7	21	3.3	2.6	2.7
25	3.3	2.45	3.4	4.3	2.95	3.9
30	4.5	4.7	4.45	4.9	4.5	4.95
			Gluc i.p.			
Time (day)						
5	0.3	0.2	0.1	0.1	0.2	0.25
10	0.15	-0.4	0.2	0.8	0.35	0.25
15	0.5	0.25	0.3	1.1	0.85	0.35

20	0.95	0.7	0.65	2.05	1.95	0.9
25	1.75	1.35	1.55	4.95	3.65	1.05
30	1.7	1.9	1.7	5	4.5	1.9
			Gluc Topical			
Time (day)						
5	0.05	0.18	0.25	0.2	0	0.2
10	0.55	0.88	-0.05	0.45	-0.6	0.95
15	0.55	1.33	0.7	0.55	-0.2	1.4
20	2	2.58	1.2	1.05	1.15	2
25	3.3	4.08	2.05	2.35	2.45	2.85
30	3.85	5.88	3	3.5	4.1	4.5
			SbL8 Topical			
Time (day)						
5	0.2	0.3	0.2	0.1	0.1	0.3
10	1.15	0.4	0.85	0.9	0.25	1.1
15	1.65	0.95	1.3	1.55	0.6	1
20	2.35	1.7	1.95	2.05	0.95	1.45
25	3.7	3.2	4.05	3.65	2.5	3.25
30	4.4	4.45	4.3	5.35	3.7	4.2
			SbL10 Topical			
Time (day)						
5	0.1	0	0.2	0	0	0.1
10	0	0.7	0	0.6	0.95	0.75
15	0.6	1.15	0.95	0.75	1.05	1.25
20	0.8	1.95	1.85	1.65	2.15	2
25	1.25	3	2.6	2.5	3.3	4.2
30	2.65	3.45	3	3	4.1	3.9

Table S8. Parasite load in the lesion of mice infected with *L. amazonensis* after different antimonial treatments (data from Figure 6b)

Parasite load in the lesion (*Leishmania* number per ng of DNA)					
Non-treated	Gluc i.p.	Gluc Topical	SbL8 Topical	Sbl10 Topical	SbL10 Oral
1155479	175071.7	359569.9	494000.2	479369.6	329230.6
561869.6	373043.3	155469.4	95670.99	319384.4	203775.1
642463.7	574903.3	326885.2	369928.9	507747.5	555340.2
809993.8	636389.8	346152.2	346190.6	666097.5	418953.1
418729.7	247976.8	608855.7	386653.4	504101.8	217939.3
440610.9	448612	640522.6	211344.3	275635.9	463645.2
